# Climate and litter traits affect the response of litter decomposition to soil fauna

**DOI:** 10.1186/s13104-023-06601-x

**Published:** 2023-11-08

**Authors:** Dangjun Wang, Wuyang Xie, Fang Yuan, Chaochao Deng, Ruimin Qin, Huakun Zhou

**Affiliations:** 1 Key Laboratory of Aquatic Botany and Watershed Ecology, Wuhan Botanical Garden, Chinese Academy of Sciences, Wuhan, 430074 China; 2 AMAP, Univ Montpellier, CIRAD, CNRS, INRAE, IRD, Montpellier, 34000 France; 3 Centre of Plant Ecology, Core Botanical Gardens, Chinese Academy of Sciences, Wuhan, 430074 China; 4 University of Chinese Academy of Sciences, Beijing, 100049 China; 5 Qinghai Provincial Key Laboratory of Restoration Ecology for Cold Region, Northwest Institute of Plateau Biology, Chinese Academy of Sciences, Xining, 810008 China

**Keywords:** Soil fauna, Terrestrial ecosystems, Climate, Functional traits, Litter decomposition

## Abstract

**Objectives:**

Soil fauna plays a crucial role in contributing to litter breakdown, accelerating the decomposition rate and enhancing the biogeochemical cycle in terrestrial ecosystems. Comprehending the specific fauna role of functional species in litter decomposition is challenging due to their vast numbers and diversity. Climate and litter quality are widely acknowledged as dominant drives of litter decomposition across large spatial scales. However, the pattern of climate and litter quality modulates the effect of soil fauna on litter decomposition remains largely unexplored. To address this gap, we conducted an extensive analysis using data from 81 studies to investigate how climate and litter traits affects soil fauna in the decomposition.

**Data description:**

The paper describes fauna body size, climate zones (tropical, subtropical and temperate), ecosystem types (forest, grassland, wetland and farmland), soil types (sand, loam and clay), decomposed duration (< 180, 180–360, > 360 days), litter initial traits, average annual temperature and precipitation. The litter traits encompass various parameters such as concentrations of carbon, nitrogen, phosphorus, potassium, lignin, cellulose, total phenol, condensed tannin, hydrolysable tannin and other nutrient traits. These comprehensive datasets provide valuable insights into the role of soil fauna on the decomposition at global scale. Furthermore, the data will give researchers keys to assess how climate, litter quality and soil fauna interact to determine decomposition rates.

## Objective

As a multifunctional heritage of plants, litter drives biogeochemical cycles and provides habitat and food resources for soil organisms [1, 2]. Its decomposition is an important ecological process that drives nutrient cycling, energy transfer and ecosystem sustainability [3, 4]. Soil fauna is an important driver of litter decomposition, soil structure stabilization, and nutrient cycling [5], but soil fauna remains relatively understudied compared to other soil organisms, such as archaea, bacteria and fungi [6]. The complexity of soil fauna species and their intricate interrelationships [5], makes it challenging to clarify contributions of individual species within the ecosystem. Many researchers have explored their ecological role in units of soil biological functional groups [7]. According to their individual size, soil fauna can be classified as small fauna (soil nematodes), medium fauna (mites, nematodes), and large fauna (earthworms, millipedes). Hence, understanding patterns of body size of soil fauna will enable us to better evaluate their contributions to the ecological processes of litter decomposition.

Climate and litter quality as pivotal factors that influence litter decomposition [8], which could explain about 60–70% of litter decomposition rates in a model [9], but the extent to which soil fauna contributes to this process remains unclear. Climate change the activity of soil fauna by altering the soil microclimate, such as litter layer temperature and humidity, thereby altering the ecosystem function and litter decomposition [10, 11]. Litter complex compounds and secondary compounds is more difficult to decompose, such as lignin, cellulose, hemicellulose, tannins and phenolics, often acting as defense mechanisms against herbivores and soil fauna attacks [12]. At the local scale, there is consensus that litter decomposition is primarily regulated by climate factors (temperature, precipitation and elevation) [8, 13], litter traits (lignin and cellulose) and soil fauna [12, 14]. However, very little is known about how climate and litter quality modulates the effect of soil fauna on litter decomposition at global scale. Our data will allow researchers to explore the relationships between environmental factors, litter initial traits, soil fauna and litter decomposition rates. To our knowledge, this dataset represents the freely available collection of data for climates, litter traits, soil fauna and litter decomposition rates from published on 81 studies (Table 1, Data file1).

## Data description

### Data collection

Data were collected from the Web of Science and the Chinese National Knowledge Infrastructure (CNKI) databases before 2022. We synthesized studies that the contribution of soil fauna on litter the decomposition in Web of Science using document search formula: TS=(decomposition* or breakdown* or processing* or decay*) and (litter* or leaf* or foliar*or trait*) and (soil fauna or soil invertebrate* or soil animal* or soil detritivore* or nematode* or earthworm* or enchytraeid* or isopod* or termite* or millipede* or tardigrade* or mite* or collembola or springtail* or snail* or gastropod* or arthropod* or protozoa* or ciliophora* or proteus* or flagellate* or coleoptera* or larvae* or insect* or spider* or scorpion* or pseudoscorpionida* or protur* or piplur* or ant* or slug* or lima* or microarthropod* or mesoarthropod* or macroarthropod* or microfauna or macrofauna or mesofauna or micro-* or meso-* or macro-*). In the China National Knowledge Infrastructure (CNKI), we used keywords such as (‘decomposition’ or ‘decay’) and (‘leaf’ or ‘litter’) and (‘soil fauna’ or ‘soil invertebrate’ or ‘soil animal’ or ‘soil detritivore’) and (‘litterbag’). This search yielded a total of 1400 references.

### Data filtering

Several criteria were used to minimise potential publication bias: (i) studies had to quantitatively compare litter mass loss, or remaining mass, or calculate the decomposing constant *k* in field litterbags experiment with different soil fauna; (ii) experiments must incorporate two data categories (soil fauna being both excluded and present treatments); (iii) soil fauna being both excluded and included must possess available data on mean, sample size or replicate, and standard deviation (SD) or standard error (SE); (iv) the published article must have at least one of the following 37 variables: litter properties of mass loss, decomposing *k*, mass remaining, concentrations of carbon, nitrogen, phosphorus, potassium, sodium, calcium, magnesium, manganese, lignin, cellulose, hemicellulose, total phenol, condensed tannins, hydrolysable tannins, leaf thickness, leaf toughness; fauna properties of specific diversity (Simpson’s diversity index and Shannon–Wiener diversity index), species evenness (Pielou’s evenness index), species richness (Margalef’s richness index), individual density, number of fauna groups; soil quality parameters including soil moisture, soil temperature, pH, soil bulk density, soil total nitrogen, soil total phosphorus, soil organic carbon, soil calcium, soil microbial biomass, soil microbial biomass carbon, soil microbial biomass nitrogen, amount of litter, fine root biomass. In total, the database covered papers published on 81 studies performed at 75 separate sites (Table 1, Data file 1).

**Table 1:** Overview of data files/data sets

Label	Name of data file/data set	File types (file extension)	Data repository and identifier (DOI or accession number)
Data file 1	Table 1	MS Excel file (.xlsx)	Science Data Bank
			10.57760/sciencedb.10380 [15]

### Limitations

(i) The climate types in this database were categorized as tropical, subtropical, and temperate zone. Tibetan Plateau belongs to the temperate zone, it may have a different climate from other temperate zones due to its elevated altitude, strong radiation and low temperature. Hence, it is advisable to consider segregating the Tibetan Plateau from the temperate zone in future research. (ii) The vegetation types in this study collected data from four distinct ecosystems: forest, grassland, wetland and farmland, but did not collect experimental data on ecosystem transition zone. The relationship between soil fauna and litter decomposition in ecological ecotones maybe differ from a single ecosystem. (iii) The methodology employed for positioning litter bag was not accurately recorded in this database, such as lay flat on the ground, the height above the ground, and the depth of burial into the ground, which may affect the experimental results.

## Data Availability

The data and methods described in this Data note can be freely and openly accessed on Science Data Bank under 10.57760/sciencedb.10380. Please see Table 1 and reference [15] for details and links to the data.
